# CCL22-Producing Resident Macrophages Enhance T Cell Response in Sjögren's Syndrome

**DOI:** 10.3389/fimmu.2018.02594

**Published:** 2018-11-08

**Authors:** Aya Ushio, Rieko Arakaki, Kunihiro Otsuka, Akiko Yamada, Takaaki Tsunematsu, Yasusei Kudo, Keiko Aota, Masayuki Azuma, Naozumi Ishimaru

**Affiliations:** ^1^Department of Oral Molecular Pathology, Tokushima University Graduate School of Biomedical Sciences, Tokushima, Japan; ^2^Department of Pathology and Laboratory Medicine, Tokushima University Graduate School of Biomedical Sciences, Tokushima, Japan; ^3^Department of Oral Medicine, Tokushima University Graduate School of Biomedical Sciences, Tokushima, Japan

**Keywords:** autoimmunity, tissue-resident macrophage, chemokine, salivary gland, Sjögren's syndrome, T cell response

## Abstract

Macrophages (MΦs) are critical regulators of immune response and serve as a link between innate and acquired immunity. The precise mechanism of involvement of tissue-resident MΦs in the pathogenesis of autoimmune diseases is not clear. Here, using a murine model for Sjögren's syndrome (SS), we investigated the role of tissue-resident MΦs in the onset and development of autoimmunity. Two unique populations of CD11b^high^ and CD11b^low^ resident MΦs were observed in the target tissue of the SS model. Comprehensive gene expression analysis of chemokines revealed effective production of CCL22 by the CD11b^high^ MΦs. CCL22 upregulated the migratory activity of CD4^+^ T cells by increasing CCR4, a receptor of CCL22, on T cells in the SS model. In addition, CCL22 enhanced IFN-γ production of T cells of the SS model, thereby suggesting that CCL22 may impair the local immune tolerance in the target organ of the SS model. Moreover, administration of anti-CCL22 antibody suppressed autoimmune lesions in the SS model. Finally, histopathological analysis revealed numerous CCL22-producing MΦs in the minor salivary gland tissue specimens of the SS patients. CCL22-producing tissue-resident MΦs may control autoimmune lesions by enhancing T cell response in the SS model. These results suggest that specific chemokines and their receptors may serve as novel therapeutic or diagnostic targets for SS.

## Introduction

Macrophages (MΦs) differentiate from bone marrow-derived monocytes or tissue-resident cells derived from yolk sac or fetal liver. These cells tend to exhibit distinct tissue-specific phenotypes, such as histiocytes in connective tissues, Kupffer cells in the liver, microglia in the central nervous system and various specialized macrophages in the alveolar, peritoneal, and synovial tissues (1, 2). Tissue-resident MΦs represent a vital component of the innate immunity system and function as phagocytic cells that engulf and digest cellular debris, foreign substances, microbes, and pathogens (3). They also secrete cytokines and chemokines that modulate the activities of other immune cells in inflammatory lesions (3). Besides phagocytosis and immune signaling, in conjunction with DCs, MΦs present antigens to T cells: this acts as a link between innate and acquired immunity (3). MΦs also contribute to the recovery or remodeling of injured tissues via promotion of angiogenesis or fibrosis (4, 5). However, the multilateral roles of tissue-resident MΦs in various inflammatory disorders are unclear.

Classically activated (M1) MΦs produce pro-inflammatory cytokines, such as interleukin (IL)-1β, interferon (IFN)-γ, and tumor necrosis factor (TNF)-α (1, 6). Alternatively activated (M2) MΦs produce anti-inflammatory cytokines, such as IL-10 and IL-4, with a specific profile that depends on the M2 MΦ subset (M2a, b, and c) (1, 7). MΦs are involved in inflammatory tissue damage associated with autoimmune response (8). In addition, MΦs also support tissue repair and restoration of immune homeostasis (9, 10). Thus, MΦs play a key role in various physiological and pathological responses as classically activated MΦs, wound-healing MΦs, and regulatory MΦs. Although tissue-resident MΦs are also considered as immune cells and have a variety of functions, the precise role of tissue-resident MΦs in autoimmune response is obscure.

Sjögren's syndrome (SS) is a chronic autoimmune disease that affects exocrine glands, such as salivary and lacrimal glands; SS also causes systemic autoimmune lesions (11, 12). A variety of mononuclear cell populations that infiltrate the salivary gland tissues were observed in patients with SS (13, 14). Among these, infiltration of T cells, B cells, DCs, and MΦs is correlated with lesion severity (15). SS is triggered by T cell-mediated autoimmune response; however, other immune cells also contribute to the onset or development of SS, including MΦs (16–18). MΦs are observed in the autoimmune lesions of salivary gland tissues of patients with SS (19). Moreover, elevated expression of MΦ-derived molecules, such as chitinase-3-like protein 1 and chitinase 1, is associated with increased severity of SS lesions, thereby indicating the involvement of MΦ in the pathogenesis of SS (20). Furthermore, several chemokines secreted from MΦs in the salivary glands of patients and animal models with SS contribute to the onset or development of SS (21, 22). However, the molecular or cellular mechanism of the pathogenesis of SS through the tissue-resident MΦs in the target organ has not been defined.

Here, using a mouse model of SS, we investigated the association of resident MΦs in the target organs of SS with the onset and development of autoimmune lesions. In particular, we evaluated the precise contribution of MΦs to T cell response in a SS model and patients with SS. The findings of the current study may help us comprehend a novel pathogenic mechanism of autoimmunity and may also help establish potential new treatments for autoimmunity.

## Materials and methods

### Mice

Female NFS/N mice carrying the mutant *sld* were bred and maintained in a specific pathogen-free mouse colony in the animal facility at Tokushima University (Tokushima, Japan). Neonatal thymectomy was performed on day 3 after birth to generate the SS model mice. Control mice used in this study were sham (non)-thymectomized NFS/*sld* mice that exhibit no inflammatory lesions in the salivary and lacrimal glands. In addition, we confirmed that the phenotypes and functions of immune cells of control mice showed no abnormality, compared with those of age- and sex-matched C57BL/6 mice. This study was conducted according to the Fundamental Guidelines for Proper Conduct of Animal Experiment and Related Activities in Academic Research Institutions under the jurisdiction of the Ministry of Education, Culture, Sports, Science and Technology of Japan. The protocol was approved by the Committee on Animal Experiments of Tokushima University and Biological Safety Research Center, Japan (Permit Number: T-27-7). All experiments were performed after administration of anesthesia, and all efforts were made to minimize suffering.

### Cell isolation

For the isolation of MΦ from the salivary gland, bilateral whole salivary gland lobes were minced into 1–3 mm pieces and were digested with collagenase (1 mg/mL, Wako), hyarulonidase (1 mg/mL, SIGMA-ALDRICH), and DNase (10 ng/mL, Roche) in Dulbecco's modified Eagle's medium (DMEM) containing 10% fetal calf serum at 37°C for 40 min using gentleMACS Dissociators (Miltenyi Biotec). Subsequently, mononuclear cells were enriched using a Histopaque-1083 (Merck) from a single-cell suspension of salivary gland tissue. Mononuclear cells were labeled with anti-CD45.2, F4/80, CD11b, CD3, and CD19 antibodies (eBioscience); subsequently, CD11b^high^ F4/80^+^ MΦs and CD11b^low^ F4/80^+^ MΦs were isolated using a cell sorter (JSAN JR Swift, Bay Bioscience). Splenocytes and cervical lymph node (cLN) cells were homogenated in DMEM containing 2% FBS using gentleMACS Dissociators (Miltenyi Biotec). Using 0.83% ammonium chloride, red blood cells were removed from the spleen cells. Splenic CD4^+^ T cells were obtained by negative selection using the EasySep mouse CD4^+^ T cell Isolation Kit (STEMCELL Technologies). Flow cytometric analysis showed that CD4^+^ cells accounted for >90% of the isolated cells. In addition, the viability of the isolated cells was checked by cell counter (CYTORECON, GE Healthcare) using trypan blue staining. The cell number was determined as the total absolute number of lymphocytes per each organ by cell counter (CYTORECON) using trypan blue staining; subsequently, the proportion of the suspended cells was analyzed by flow cytometry. The absolute number of T cells or macrophages was calculated using the data pertaining to total cell number and the proportion. As for the salivary gland, we used bilateral lobes to determine the cell number and the proportion of immune cells. As for splenocytes and cervical lymph node cells, the whole spleen and bilateral cervical lymph nodes per mouse were used to determine the cell number and the proportion.

### Flow cytometric analysis

Immune cells were stained using antibodies against FITC-conjugated anti-mouse CD206 (BioLegend, C068C2) and CD11c (eBioscience, N418) mAbs, PE-conjugated anti-mouse MHC class II (Miltenyi Biotec, REA478), CD86 (BD Bioscience, GL1), CD204 (eBioscience, M204PA), CCR2, CX3CR1, CCR4 (BioLegend, SA203G11, SA011F11, and 2G12), PE-Cy5.5-conjugated anti-mouse CD3 and CD19 (TONBO Biosciences, 145-2C11, and 6D5) and 7-Aminoactinomycin D (7-AAD) staining solution (TOMBO Biosciences), PE-Cy7-conjugated anti-mouse CD11b (TONBO Biosciences, M1/70), APC-conjugated anti-mouse F4/80 and CD36 (BioLegend, BM8 and HM36), and APC-Cy7-conjugated anti-mouse CD45.2 (TOMBO, 104) mAbs. For detecting intracellular CCL22 expression, rabbit anti-CCL22/MDC (abcam, rabbit monoclonal IgG, EPR1362) Ab, and Alexa Fluor 568 goat anti-rabbit IgG (Invitrogen) were used. A FACScant flow cytometer (BD Biosciences) was used to identify the cell populations according to expression profile. Viable cells were checked by gating on side scatter (SSC)/forward scatter (FSC), FSC-H/FSC-A, 7AAD, CD45.2, and CD4. We used 5 × 10^5^ cells as a sample for the analysis. Data were analyzed using the FlowJo FACS Analysis software (Tree Star Inc.).

### Phagocytosis assay

Phagocytosis was assessed for using the Phagocytosis Assay Kit (IgG FITC, Cayman Chemical). Mononuclear cells from the salivary glands provided as previously described were cultured in DMEM containing 10% FBS at 37°C and were washed with PBS 24 h later to remove unbounded cells. Adherent cells were incubated with the opsonized beads for 2 h at 37°C or at 4°C for controls; this was followed by washing with PBS. The phagocytic activity of F4/80^+^ CD11b^high^ and F4/80^+^ CD11b^low^ MΦs was analyzed using flow cytometry.

### RNA extraction

Total RNA was isolated from the purified macrophages and cultured cells using the RNeasy Plus Mini Kit (Qiagen) with a gDNA eliminator column treatment step. Total RNA was extracted from the salivary glands, lung, spleen, and liver tissues using Isogen (FUJIFILM Wako Pure Chemical). Total RNA was then reverse-transcribed into cDNA using the PrimeScript II reverse transcriptase (Takara Bio Inc).

### Quantitative reverse transcription-polymerase chain reaction (qRT-PCR)

Expression levels of mRNAs encoding CCL22, CCR4, IFN-γ, IL-4, IL-17, and β-actin were determined using a 7300 real time PCR system (Applied Biosystems) with TB Green Premix Ex Taq II (Takara Bio). PCR was performed followed by 40 cycles for 10s at 95°C and for 15 s at 60°C. The primer sequences used were as follows: CCL22: forward, 5′-TCATGGCTACCCTGCGTGTC-3′, and reverse, 5′-CCTTCACTAAACGTGGCAGAG-3′, CCR4: forward, 5′-GGCTACTACGCCGCCGAC-3′, and reverse, 5′-TACCAAAACAGCATGATGCC-3′, IFN-γ: forward, 5′-AGCGGCTGACTGAACTCAGATTGTA-3′, and reverse, 5′-GTCACAGTTTTCAGCTGTATAGGG-3′, IL-4: forward, 5′-TCTCATGGAGCTGCAGAGACTCT-3′, and reverse, 5′-TCCAGGAAGTCTTTCAGTGATGTG-3′, IL-17: forward, 5′-AGTGTTTCCTCTACCCAGCAC-3′, and reverse, 5′-GAAAACCGCCACCGCTTAC-3′, β-actin: forward, 5′-GTGGGCCGCTCTAGGCACCA-3′, and reverse, 5′-CGGTTGGCCTTAGGGTTCAGGGGG-3′. Relative mRNA expression of each transcript was normalized against β-actin mRNA.

### Histological analysis

Salivary gland tissues were fixed with 10% phosphate-buffered formaldehyde (pH 7.2), and were prepared for histological examination. Sections (4 μm) were stained with hematoxylin and eosin (H&E).

### Immunohistochemistry

Tissue sections (6 μm) were deparaffinized in xylenes and were rehydrated by passage through serial dilutions of ethanol in distilled water. Heat-induced antigen retrieval was performed in Immunoactive (Matsunami Glass Ind. Ltd) with microwave thrice for 5 min. Anti-mouse F4/80 antibody (eBioscience), anti-human CCL22 (abcam), and anti-mouse CCL22 (abcam) antibody were applied to the sections; the sections were then incubated overnight at 4°C. After washing with PBS, the sections were incubated with biotinylated second antibody and horseradish peroxidase (HRP)-conjugated streptavidin solution (DAKO). HRP reacted with the 3,3′-diaminobenzidine (DAB) substrate using the Histofine DAB substrate kit (Nichirei Biosciences Inc.). The sections were counterstained with hematoxylin.

### Confocal analysis

Frozen sections (6 μm) of salivary gland tissues were fixed with cold acetone, blocked with 10% goat serum (DAKO), and then stained with a rabbit monoclonal antibody against CCL22/MDC (abcam), FITC-conjugated anti-mouse F4/80 (BioRad), rat monoclonal antibody against anti-EpCAM (eBioscience, G8.8), anti-CD3 (eBioscience, 1.45-2C11), anti-CD19 (eBioscience, eBio1D3), and biotinylated anti-CD11c (Biolegend, N418) antibodies. After washing with PBS, Alexa Fluor 568-conjugated anti-rabbit IgG (Invitrogen) and Alexa Fluor 488-conjugated anti-fluorescein Green goat IgG fraction (Invitrogen), or Alexa Fluor 488-conjugated anti-rat IgG (Invitrogen), or Alexa Fluor 488-conjugated streptavidin (Invitrogen) were used as secondary antibodies. Nuclear DNA was stained with 4′,6-Diamdino-2-phenylindole dihydrochloride (DAPI) (Invitrogen). In addition, the paraffin-embedded sections from SS patients and controls were stained with anti-CD68 (DAKO, Klon EBM11), anti-Keratin (DAKO, AE1/AE3), anti-CD3 (DAKO, F7.2.38), anti-CD19 (DAKO, LE-CD19), anti-S100 (abcam, 4C4.9), and anti-CCL22 Abs (abcam). The sections were examined using a PASCAL confocal laser-scanning microscope (LSM: Carl Zeiss) at 400 × magnification. LSM image browser version 3.5 (Carl Zeiss) was used for image acquisition.

### Gene-expression analysis with PCR array

Chemokine-related gene expression of sorted CD11b^low^ and CD11b^high^ sMΦs were analyzed using PCR array. Total RNA was reverse-transcribed to cDNAs using the RT^2^ First Strand Kit (Qiagen). The cDNA was applied to RT2 Profiler PCR array (PAMM-022ZE-1) plates to detect the expression of genes related to chemokines. Real-time PCR reactions were performed on a 7900HT Real-Time PCR System. (Applied Biosystems). Raw data were extracted and analyzed according to the Qiagen RT^2^ Profiler PCR Array Data Analysis web portal. Gene expression levels were calculated using the ΔΔCt method; the relative gene expression levels were normalized using four house-keeping genes (*Actb, B2m, Gapdh*, and *Gusb*).

### Chemotactic migration assay and *in vitro* culture of CD4^+^ T cell with CCL22

Splenic CD4^+^ T cell were cultured for 5 days in RPMI 1640 containing 10% FBS with Dynabeads mouse T-activator CD3/CD28 (Invitrogen) at bead/cells ratio of 1:1 and 30 U/mL of rIL-2 (eBioscience). After serum starvation in RPMI 1640 medium for 3 h, splenic CD4^+^ T cells were seeded (1.0 × 10^6^ cells in 400 μL) in Millicell Culture Plates Inserts (5.0-μm pore size, Merck Millipore). 600 μl of RPMI 1640 containing 0.1% BSA containing CCL22 (200 ng/mL; R&D Systems Inc.) was added to the lower chamber. The cells were cultured for 3 h at 37°C and then the numbers of migrated cells were counted by cell counter (CYTORECON, GE Healthcare UK).

CD4^+^ T cells purified from the spleen, cLNs, and salivary glands in SS model mice were cultured with CCL22 for 6 h. Then the mRNA of the T cells was purified for analysis of cytokine gene expression.

### Analysis of intracellular cytokine expression

CD4^+^ T cells (1 × 10^6^/well) isolated form spleen of control and SS model mice, or T cells (2 × 10^5^/well) isolated form salivary gland tissues of SS model mice were stimulated with Dynabeads mouse T-activator CD3/CD28 (Invitrogen) at bead/cell ratio of 1:4 and CCL22 (200 ng/mL) for 48 h, and were then cultured with phorbol myristate acetate (PMA; 50 ng/mL, Sigma-Aldrich, St. Louis, MO) and ionomycin (IM; 1 μg/mL, Sigma-Aldrich) in the presence of Brefeldin A (eBioscience) for the last 6 h. After washing, cells were stained with an anti-CD4 mAb, fixed in fixation/permeabilization solution (eBioscience); permeabilized in permeabilization buffer (eBioscience); and stained with anti-IFN-γ (eBioscience, XMC1.2), IL-4 (TONBO, 11B11), and IL-17A (eBioscience, TC11-18H10.1).

### Analysis of cytokine levels

CD4^+^ T cells isolated from salivary gland tissues of SS model mice were cultured with or without CCL22 (200 ng/mL) in the presence of Dynabeads mouse T-activator CD3/CD28 (Invitrogen) at bead/cell ratio of 1:4 for 48 h and subsequently cultured with PMA (50 ng/mL, Sigma-Aldrich) and IM (1 μg/mL, Sigma-Aldrich) for the last 6 h. Cytokine levels, including IFN-γ, IL-4, and IL-17, in the supernatant were measured using a Cytokine 20-Plex Mouse Panel Luminex assay kit (Invitrogen) according to the manufacturer's instructions.

### Administration of anti-CCL22 antibody

Four μg of goat anti-CCL22 polyclonal Ab (R&D System) or control polyclonal goat IgG Ab (Santa Cruz Biotechnology, sc-3887) was intravenously injected into the SS model mice aged 8 weeks on alternate days for 2 weeks. The mice were examined at 10 weeks of age.

### Human subjects

This study was approved by the Institutional Review Board of the Tokushima University Hospital, Japan (No. 2802). All patients with SS were diagnosed according the criteria for diagnosis of SS by the Japanese Ministry of Health and the American College of Rheumatology. Labial salivary gland (LSG) samples were obtained from patients with SS and controls. The degree of lymphocytic infiltration in the specimens was determined using a modification of the system originally introduced by Greenspan (23). The results are classified into five grades in a blind manner by three pathologists: Grade 0 = the absence of lymphocytes and plasma cells per 4 mm^2^ in LSGs, Grade 1 = mild infiltration of lymphocytes and plasma cells per 4 mm^2^ in LSGs, Grade 2 = a moderate infiltration or less than one focus per 4 mm^2^ in LSGs, Grade 3 = a single focus per 4 mm^2^ in LSGs, and Grade 4 = more than one focus per 4 mm^2^ in LSGs. One focus refers to an aggregate of ≧50 mononuclear cells, including lymphocytes, histiocytes, and plasma cells around the ductal structure. Five to seven samples per group were used for analysis. Control samples were collected from non-inflamed tissues of patients with mucous cyst or other oral disorders.

### Statistical analysis

Differences between individual groups were determined using two-tailed Student's *t*-test or between more than two groups using one-way ANOVA with Turkey's multiple comparison post-test. *p* < 0.05 was considered statistically significant. Power calculations were performed before the beginning of the experiments to determine the sample size for experiments using human samples or animals. Data are presented as mean ± standard error of mean (SEM).

### Data availability

The PCR array data are available from the Gene Expression Omnibus database under accession number GSE110816.

## Results

### MΦs in the salivary gland of the SS model mouse

**Figure 1:**
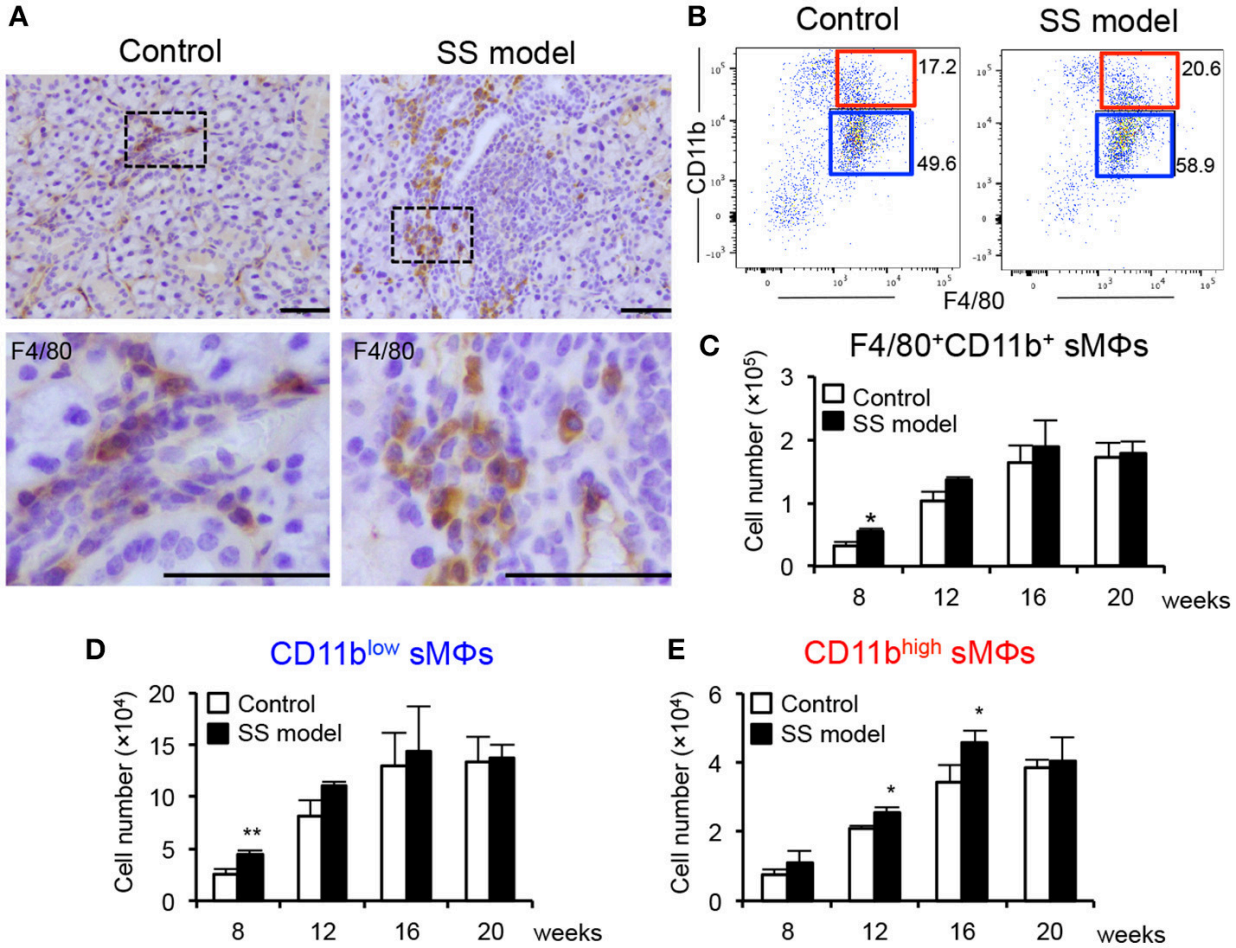
Salivary gland macrophages (sMΦs) of the SS model mice. **(A)** Immunohistochemical analysis of F4/80^+^ macrophages in the salivary gland sections obtained from the controls and SS models at 8 weeks of age. Photos are representative of 5 mice in each group. Scale bar: 50 μm **(B)** CD11b^high^ and CD11b^low^ F4/80^+^ sMΦs in the controls and SS models. **(C)** Total number of F4/80^+^ sMϕs in the controls and SS models from 8 to 20 weeks of age. Bilateral whole salivary gland lobes per mouse were minced and enzymatically digested. Mononuclear cells were enriched using a Histopaque-1083. Cell number was determined by cell counter using trypan blue staining and flow cytometer with antibodies for specific markers. **(D)** Number of CD11b^low^ F4/80^+^ sMΦs in the controls and SS models from 8 to 20 weeks of age. **p < 0.005 by Student's *t*-test. **(E)** CD11b^high^ F4/80^+^ sMΦs in the controls and SS models from 8 to 20 weeks of age. Data are presented as the mean ± SEM. *p < 0.05 by Student's *t*-test. *n* = 5–10 **(C–E)**.

We have established a mouse model of SS wherein NFS/*sld* mice are thymectomized on day 3 after birth (24, 25). The autoimmune lesions in the salivary and lacrimal glands are observed from 6 weeks of age (24). The main subset of immune cells infiltrated in the target organ of the SS model mice at 6 weeks of age is CD4^+^ T cells; small populations of CD8^+^ T cells, B cells, macrophages (MΦs), and dendritic cells are also observed (26). The proportion of infiltrated immune cells in the target organ changes with age. At 8 weeks of age, the autoimmune lesions are observed in almost 100% of the SS model mice (24). Female mice exhibit faster onset of disease and more severe inflammatory lesions compared with male mice. In addition, autoantibodies such as anti-SSA, anti-SSB, and anti-α-fodrin were detected in the SS model (24, 25). We compared the distribution of MΦs in the salivary glands (sMΦs) of control and SS model mice. Immunohistochemical analysis revealed sporadic F4/80^+^ MΦs around ductal, acinar cells and small vessels in the control mice (Figure 1A). By contrast, many F4/80^+^ MΦs were observed surrounding the autoimmune lesions in the SS model mice at 8 weeks of age (Figure 1A).

Next, the surface markers on sMΦs were analyzed using flow cytometry. CD45.2^+^ CD3^−^ CD19^−^-gated monocytes (Supplemental Figure 1) were examined for CD11b and F4/80 expression (Figure 1B). Two subsets that displayed CD11b^high^ F4/80^+^ and CD11b^low^ F4/80^+^ MΦs were detected in the salivary glands (Figure 1B). These two subsets were evaluated using control and SS model mice from 8 to 20 weeks of age. At 8 weeks of age, the number of F4/80^+^ CD11b^+^ sMΦs in the SS model mice was significantly higher than that in control mice (Figure 1C). Although the cell numbers in both the control and SS model mice increased with age, there was no difference in the number between control and SS model from 12 to 20 weeks of age (Figure 1C). At 8 weeks of age, the number of CD11b^low^ F4/80^+^ sMΦs in the SS model mice was significantly higher than that in the control mice; however, no changes were observed between the control and SS model mice from 12 to 20 weeks of age (Figure 1D). In contrast, the number of CD11b^high^ F4/80^+^ sMΦs in the SS model mice aged between 12 and 16 weeks was significantly higher than that in the control mice (Figure 1E). At 20 weeks, there was no difference in the number of CD11b^high^ F4/80^+^ sMΦs between control and SS model mice (Figure 1E). These findings indicate the existence of CD11b^high^ and CD11b^low^ F4/80^+^ sMΦ subsets; these two subsets may play a role in the onset or development of autoimmune lesions in the target organs of SS model mice.

### Phenotype and function of two MΦ subsets in salivary glands

**Figure 2:**
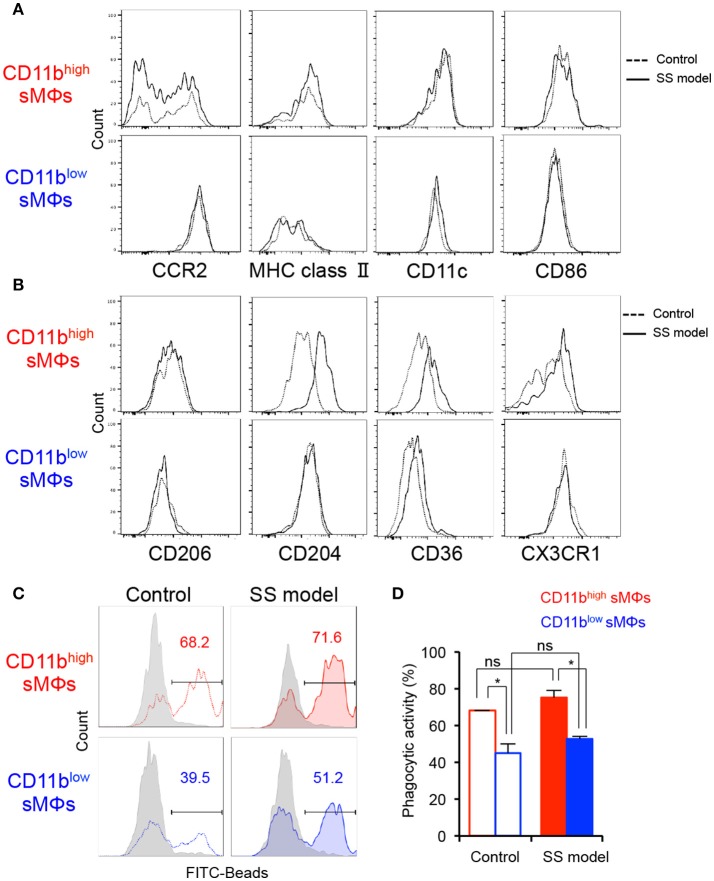
Phenotypes and functionality of tissue-resident MΦs in salivary glands of controls and SS models. **(A,B)** Key surface markers on CD11b^high^ and CD11b^low^ sMΦs of control and SS model mice were detected using flow cytometry. Data are representative of 5 mice in each group at 12 weeks of age. **(C)** Phagocytic activity of CD11b^high^ and CD11b^low^ sMΦs of control and SS model mice at 12 weeks of age was determined by *in vitro* assay with FITC-conjugated beads. Data are representative of three independent experiments. Gray shadow: 4°C culture. **(D)** Phagocytic activity is shown as mean ± SEM. *n* = 4–6 per group. *p < 0.05 by Student's *t*-test.

To define the difference between the cell surface phenotypes of the two subsets in the salivary glands, key MΦ markers were analyzed using flow cytometry. No significant differences were observed between the control and SS model mice at 12 weeks of age with respect to any of the markers of two subsets (Figure 2A). Among M1 MΦ markers, including CCR2, MHC class II, CD11c, and CD86, the expressions of MHC class II, CD11c, and CD86 on CD11b^high^ F4/80^+^ sMΦs were higher than those on CD11b^low^ F4/80^+^ sMΦs (Figure 2A). The expression pattern of CCR2 and CX3CR1 expression on CD11b^high^ F4/80^+^ sMΦs suggested that a fraction of these cells carried the chemokine receptor including CCR2 or CX3CR1, whereas the other fraction did not (Figures 2A,B). Expression of CD206 (a M2 and tissue-resident MΦ marker) on CD11b^high^F4/80^+^ sMΦs was enhanced compared with that on CD11b^low^F4/80^+^ sMΦs (Figure 2B). In addition, the expressions of CD204 and CD36 (scavenger receptors) on CD11b^high^ F4/80^+^ sMΦs in the SS model were higher than the control mice (Figure 2B). These findings indicate that CD11b^low^ F4/80^+^ sMΦs are M1-like MΦ whereas CD11b^high^ F4/80^+^ sMΦs are similar to the phenotype of M2-like and tissue resident-like MΦs. However, it is possible that the sMΦs may differentiate into the phenotype or function independent of their differentiation into M1 and M2 MΦs.

To evaluate the *in vitro* phagocytic activity of the two subsets, sMΦs of the control and SS model mice were analyzed using FITC-labeled latex beads. With respect to the phagocytic activity of CD11^high^ F4/80^+^ sMΦs, no significant differences were observed between the control and SS model mice (Figures 2C,D). There was no difference in the phagocytic activity of CD11b^low^ F4/80^+^ sMΦs between the SS model and control mice (Figures 2C,D). Contrarily, in both control and SS model mice, the phagocytic activity of CD11^high^ F4/80^+^ sMΦs was significantly higher than that of the CD11b^low^ F4/80^+^ sMΦs (Figures 2C,D). These findings indicate that the two subsets of MΦs in the salivary gland are functionally distinct.

### Chemokine expression of CD11b^high^ F4/80^+^ sMΦs in the SS model mice

**Figure 3:**
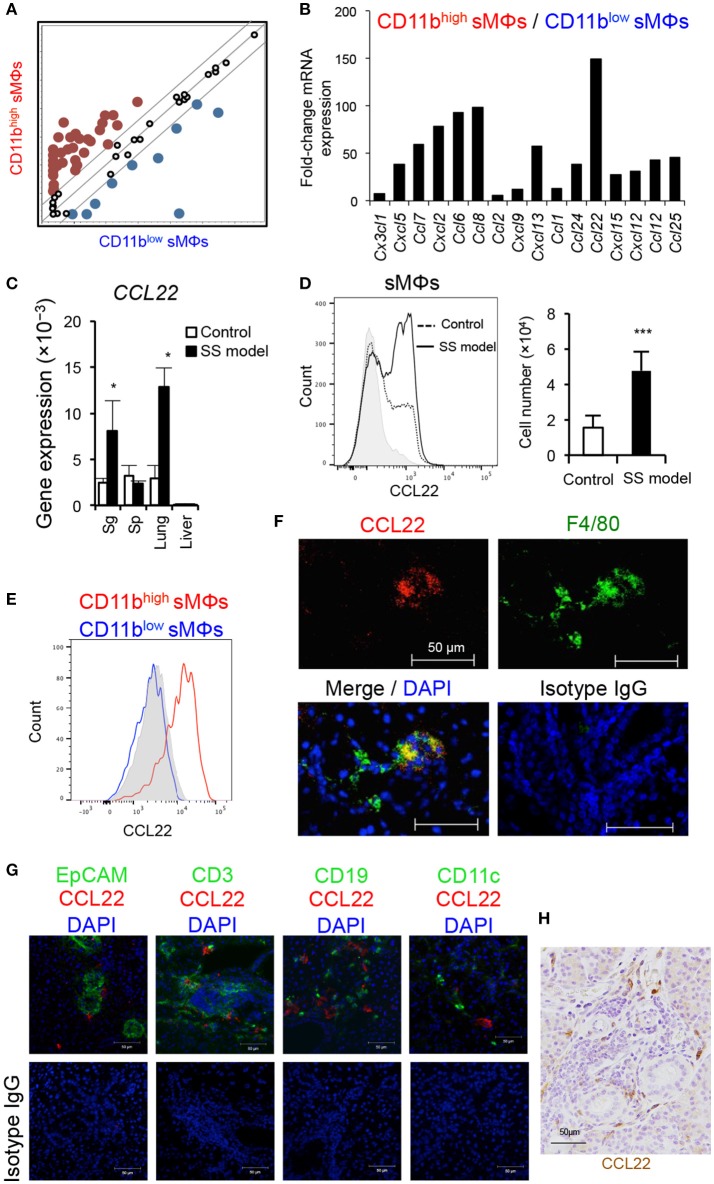
Comprehensive analysis of chemokine genes of sMΦs. **(A,B)** Chemokine mRNA expressions of isolated CD11b^high^ and CD11b^low^ sMΦs of the SS model mice at 12 weeks of age were analyzed using PCR-array. Up-and downregulated genes of CD11b^high^ sMΦs compared with CD11b^low^ sMΦs of the SS model mice. The lines indicate 5-fold change in the gene expression. Red dots are upregulated genes, and blue dots are downregulated genes. Data are representative of three independent experiments. **(C)** CCL22 mRNA expression levels of various tissues of the SS model and control mice at 12 weeks of age were determined using qRT-PCR. Data are presented as mean ± SEM. *p < 0.05 by Student's *t*-test. *n* = 5. **(D)** CCL22 expression of sMΦs in salivary glands of control and SS model mice (left). CCL22 positive cell number (right). Data are representative of five mice in each group and are presented as mean ± SEM. ***p < 0.0005 by Student's *t*-test. *n* = 5. Gray shadow is isotype control. **(E)** Comparison of CCL22 expression between CD11b^high^ and CD11b^low^ sMΦs of the SS model mice at 12 weeks of age. Data are representative of five mice. Gray shadow is isotype control. **(F)** CCL22-producing sMΦs of the SS model mice were detected by confocal microscopic analysis. Data are representative of five mice. **(G)** Confocal microscopic analysis of CCL22 expression of EpCAM^+^ epithelial cells, CD3^+^ T cells, CD19^+^ B cells, and CD11c^+^ DCs in the salivary gland tissues form SS model mice. Data are representative of three mice. Nuclei were stained with DAPI. **(H)** Immunohistochenical analysis of CCL22 expression using the salivary gland tissues from SS model mice. The result is representative of three mice. Nuclei were stained with hematoxylin.

Next, to define the role of the two subsets of sMΦs in the formation of autoimmune lesions, we focused on chemokine gene expression of sMΦs of the SS model mice. Using PCR-array, we comprehensively compared chemokine mRNA gene expression between CD11b^high^ and CD11b^low^ F4/80^+^ sMΦs in the SS model mice (Figure 3A). Relative to that of CD11b^low^ sMΦs, over 50-fold increase was observed in the mRNA expression of CD11b^high^ sMΦs; this increase was observed in several genes, such as *CCL7, CXCL2, CCL6, CCL8, CXCL13*, and *CCL22* (Figure 3B). Among these, *CCL22* mRNA expression level of CD11b^high^ sMΦs was over 150-times higher than that of CD11b^low^ sMΦs (Figure 3B). In addition, quantitative RT-PCR assay revealed that *CCL22* mRNA level was significantly higher in salivary gland tissues of the SS model mice than of the control mice (Figure 3C). Moreover, the *CCL22* mRNA level was also significantly higher in lung tissues of the SS model mice than of the control mice (Figure 3C). In addition, intracellular flow cytometric analysis revealed significantly higher expression of CCL22 in the sMΦs in the SS model mice than in the control mice (Figure 3D). Furthermore, CD11b^high^ F4/80^+^ but not CD11b^low^ F4/80^+^ sMΦs strongly expressed CCL22 in the SS model mice (Figure 3E). Moreover, CCL22-producing F4/80^+^ sMΦs were observed in the SS model mice by confocal microscopic analysis (Figure 3F). By contrast, confocal microscopic analysis indicated that EpCAM^+^ epithelial cells, CD3^+^ T cells, CD19^+^ B cells, and CD11c^+^ DCs did not express CCL22 (Figure 3G). Immunohistochemical analysis showed that interstitial cells such as fibroblasts, endothelial cells, and nerve cells did not also express CCL22 (Figure 3H). The results suggest that CCL22-producing sMΦs may play a potent role in the pathogenesis of SS.

### Contribution of CCL22 to migration and cytokine production of T cells

**Figure 4:**
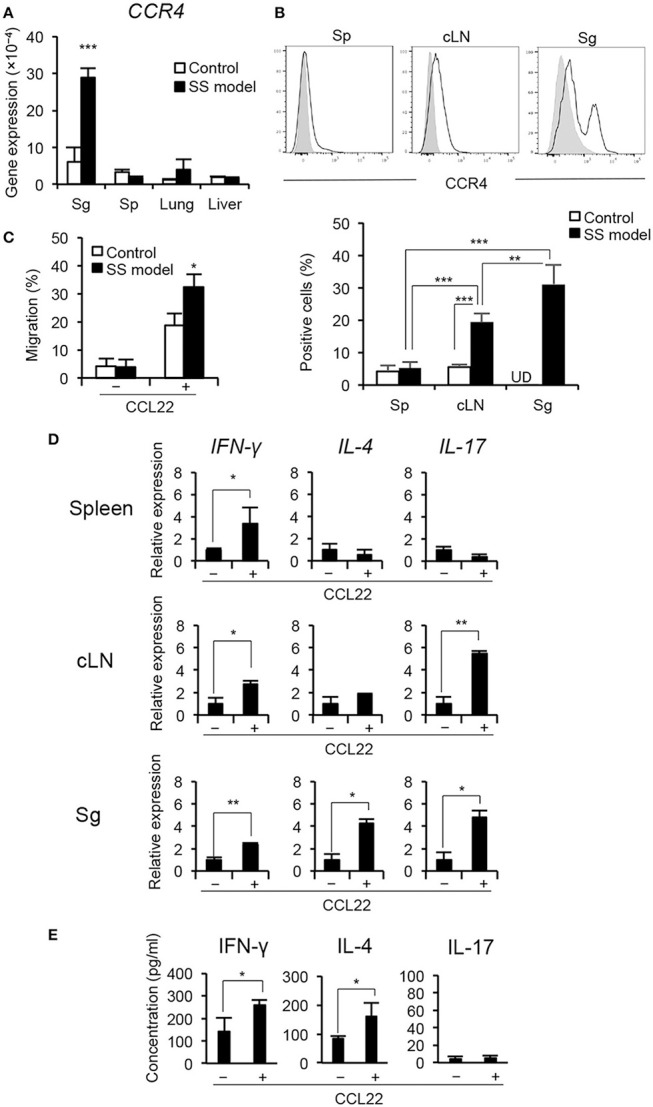
CCR4 expression on peripheral T cells and migratory activity of T cell by CCL22. **(A)** CCR4 mRNA expression levels in tissues were determined by qRT-PCR. Data are presented as mean ± SEM. n = 5. **(B)** CCR4 expression on CD4^+^ T cells purified form the spleen (Sp), cLN, and salivary glands (Sg) of SS model mice at 12 weeks of age was determined by flow cytometric analysis. Data are representative of five mice, and mean ± SEM of CCR4^+^ cells (%) of CD4^+^ T cells. Gray shadow is isotype control. **p < 0.005, ***p < 0.0005 by one-way ANOVA with Turkey's multiple comparison post-test. UD, undetermined. **(C)** Migratory activity of CD4^+^ T cells from SS model mice to CCL22 (200 ng/mL) was analyzed by *in vitro* migration assay using trans-well. Data are representative of three independent experiments. *p < 0.05 by Student's *t*-test. **(D)** CD4^+^ T cells purified from the spleen, cLNs, and salivary glands in SS model mice at 12 weeks of age were cultured with CCL22 for 6 h. The mRNA expressions of IFN-γ, IL-4, and IL-17 were detected by qRT-PCR. Data are presented as mean ± SEM relative to that without CCL22 and are representative of three independent experiments. *p < 0.05, **p < 0.005 by Student's *t*-test. **(E)** CD4^+^ T cells isolated from salivary gland tissues of SS model mice were cultured with or without CCL22 (200 ng/mL) in the presence of anti-CD3/CD28 mAb-beads for 48 h, and subsequently cultured with PMA (50 ng/mL) and iomomycin (IM) (1 μg/mL) for the last 6 h. Cytokine levels, including IFN-γ, IL-4, and IL-17, in the supernatant were measured using a Cytokine 20-Plex Mouse Panel Luminex assay kit. Data are expressed as the mean concentration pg/mL) ± SEM, n = 4 per group. *p < 0.05 by Student's *t*-test.

We evaluated the expression of CCR4 (a receptor of CCL22) in the lymphoid organs and the target organs of control and the SS model mice. The expression level of *CCR4* mRNA was significantly higher in the salivary glands of the SS model mice than of the control mice (Figure 4A). The expression in the salivary glands of SS model mice was considerably higher than that of the spleen (Sp), lung, and liver (Figure 4A). Furthermore, the expression of CCR4 on CD4^+^ T cells in spleen, cervical lymph node (cLN), and salivary glands was assessed using flow cytometry. The proportion of CCR4^+^ CD4^+^ T cells was significantly higher in the salivary glands than in the spleen and cLN of the SS model mice (Figure 4B). There was no significant difference in the proportion of CCR4^+^ CD4^+^ T cells between spleen and cLN in control mice (Figure 4B). We analyzed the mRNA expressions of the other chemokine receptors, such as *CXCR3A, CCR3*, and *CX3CR1* mRNA in the salivary gland tissues of control and SS model mice. *CXCR3A* mRNA expression in the salivary gland tissues from SS model mice was significantly higher than that in the salivary gland tissues from control mice (Supplemental Figure 2A). In addition to CXCR4, other chemokine receptors may affect the pathogenesis of SS. Next, an *in vitro* migration assay was performed using CD4^+^ T cells purified from spleen cells to evaluate the migratory activity of T cells toward CCL22. The migratory activity of CD4^+^ T cells purified from the spleen of the SS model mice was significantly higher than that of CD4^+^ T cells purified from the spleen of the control mice (Figure 4C, Supplemental Figure 2B). These findings indicate that T cell migration is controlled by CCL22 in the SS model mice.

CCL22 also plays a key role in T cell differentiation in addition to T cell migratory activity (27). In this SS mouse model, Th1 cells that produce IFN-γ contribute to the pathogenesis of autoimmune lesions in the target organ (24) (Supplemental Figure 3A). Splenic CD4^+^ T cells from the control and SS model mice were cultured with CCL22 for 6 h; mRNA expression of cytokines, including *IFN-*γ, *IL-4*, and *IL-17*, was then determined by qRT-PCR. IFN-γ mRNA expression of CD4^+^ T cells purified from the spleen of the SS model mice was significantly enhanced in response to CCL22 (Figure 4D). *IL-17* mRNA of splenic CD4^+^ T cells in the control mice was significantly enhanced in response to CCL22, whereas no change was observed in *IFN-*γ and *IL-4* mRNA (Supplemental Figure 3B). Further, besides up-regulated *IFN-*γ mRNA, *IL-17* mRNA of CD4^+^ T cells from the cLN in the SS model mice was significantly increased in response to CCL22 (Figure 4D). Finally, mRNA expressions of all cytokines in CD4^+^ T cells obtained from the salivary glands of the SS model mice were significantly increased in response to CCL22 (Figure 4D). Moreover, to examine whether CCL22 influences the cytokine secretion by T cells, concentrations of IFN-γ, IL-4, and IL-17 were analyzed using the culture supernatant of anti-CD3/CD28-stimulated CD4^+^ T cells isolated from the salivary gland tissues of SS model mice. Concentrations of IFN-γ and IL-4 were significantly enhanced by CCL22, whereas no changes were observed in IL-17 level (Figure 4E). In addition, to examine the protein level of cytokine production by CCL22, intracellular expressions of IFN-γ, IL-4, and IL-17 in the presence of CCL22 were analyzed using anti-CD3/CD28-stimulated CD4^+^ T cells from spleen of control and SS model mice. IFN-γ expression in CD4^+^ T cells of SS model mice was significantly enhanced by CCL22, whereas there were no changes in IL-4 and IL-17 expressions (Supplemental Figures 3C,D). These findings indicate that CCL22 may disturb the regulation of IFN-γ production by T cells in the target organ.

### Therapeutic effect of anti-CCL22 antibody (Ab) administration on autoimmune lesions in the SS model

**Figure 5:**
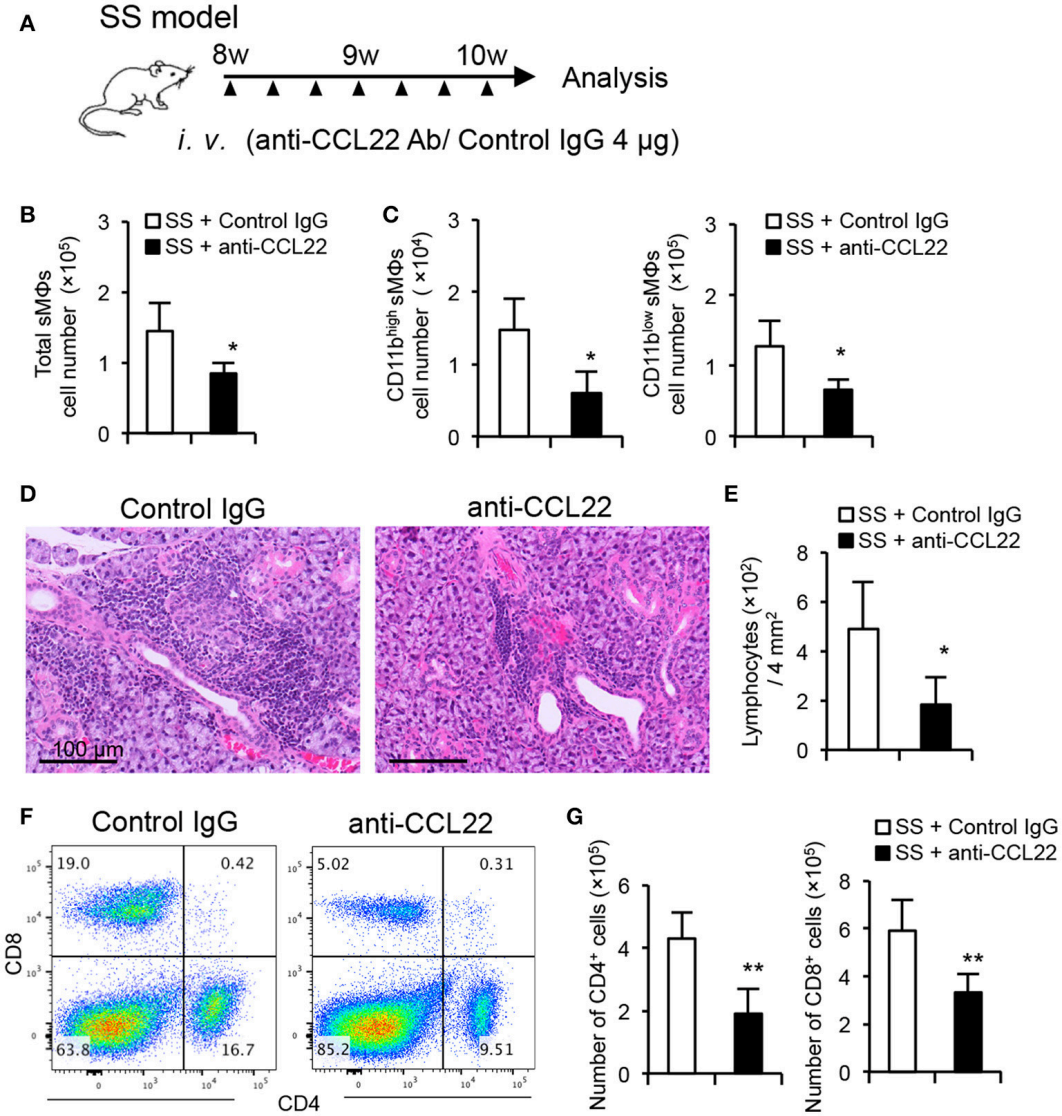
Therapeutic effect of anti-CCL22 Ab administration on autoimmune lesions in SS model mice. **(A)** Experimental schedule. Anti-CCL22 Ab (4 μg) was intravenously injected into a mouse on alternate days for 2 weeks. **(B)** Total number of sMΦs infiltrated in the salivary gland tissues from control IgG and anti-CCL22 Ab treatment was determined by cell counter and flow cytometry. **(C)** The number of CD11b^low/high^ sMΦs in the salivary gland tissues was determined using cell counter and flow cytometry. **(D)** Pathological analysis was performed using H&E-stained sections of salivary gland tissues from the SS model mice administered with anti-CCL22 Ab and isotype control antibody. Data are representative of five mice in each group. **(E)** Number of lymphocytes infiltrated into salivary gland tissues within 2 × 2 mm^2^ was counted using H&E-stained sections. **(F)** CD4/CD8 T cell phenotype in the salivary gland tissues was analyzed by flow cytometry. Data are representative of five mice in each group. **(G)** The number of CD4^+^ (left) and CD8^+^ T cells in the salivary gland tissues was determined using cell counter and flow cytometry. Data are presented as mean ± SEM of five mice (B, C, E, and G). *p < 0.05 **p < 0.005 by Student's *t*-test.

SS model mice were administered with anti-CCL22 Ab from 8 to 10 weeks of age to determine the effect of CCL22 inhibition on autoimmune lesions. Anti-CCL22 Ab (4 μg) was intravenously injected into a mouse on alternate days for 2 weeks (Figure 5A). After treatment with anti-CCL22 Ab, the number of F4/80^+^ CD11b^+^ total sMΦs in salivary glands of SS model mice was significantly decreased compared with that of SS model mice treated with control IgG (Figure 5B). Furthermore, the number of both macrophages, CD11b^high^ and CD11b^low^ F4/80^+^ sMΦs, in salivary glands of anti-CCL22-treated mice was significantly decreased compared with that of control IgG-treated SS model mice (Figure 5C), suggesting that other cells would be activated by secreting CCL22. Pathological examination revealed that when compared with the effect of isotype control Ab on SS model mice, anti-CCL22 Ab considerably suppressed the inflammatory lesions in the salivary glands of the SS model mice (Figure 5D). The number of lymphocytes infiltrated in the salivary gland tissue (/4 mm^2^) was significantly lower in the anti-CCL22 Ab-treated mice than in the control isotype antibody-treated mice (Figure 5E). In addition, flow cytometric analysis revealed that the number of CD4^+^ cells that had infiltrated in the salivary glands was significantly lower in the SS model mice injected with anti-CCL22 Ab was significantly decreased compared with that of SS model mice injected with isotype control Ab (Figures 5F,G). As for CD8^+^ T cells, the decrease of the proportion and the cell number was observed in anti-CCL22 Ab-treated mice (Figures 5F,G). These results suggest that CCL22-producing sMΦs may serve as a target for treating SS.

### Detection of CCL22-producing sMΦs in patients with SS

**Figure 6:**
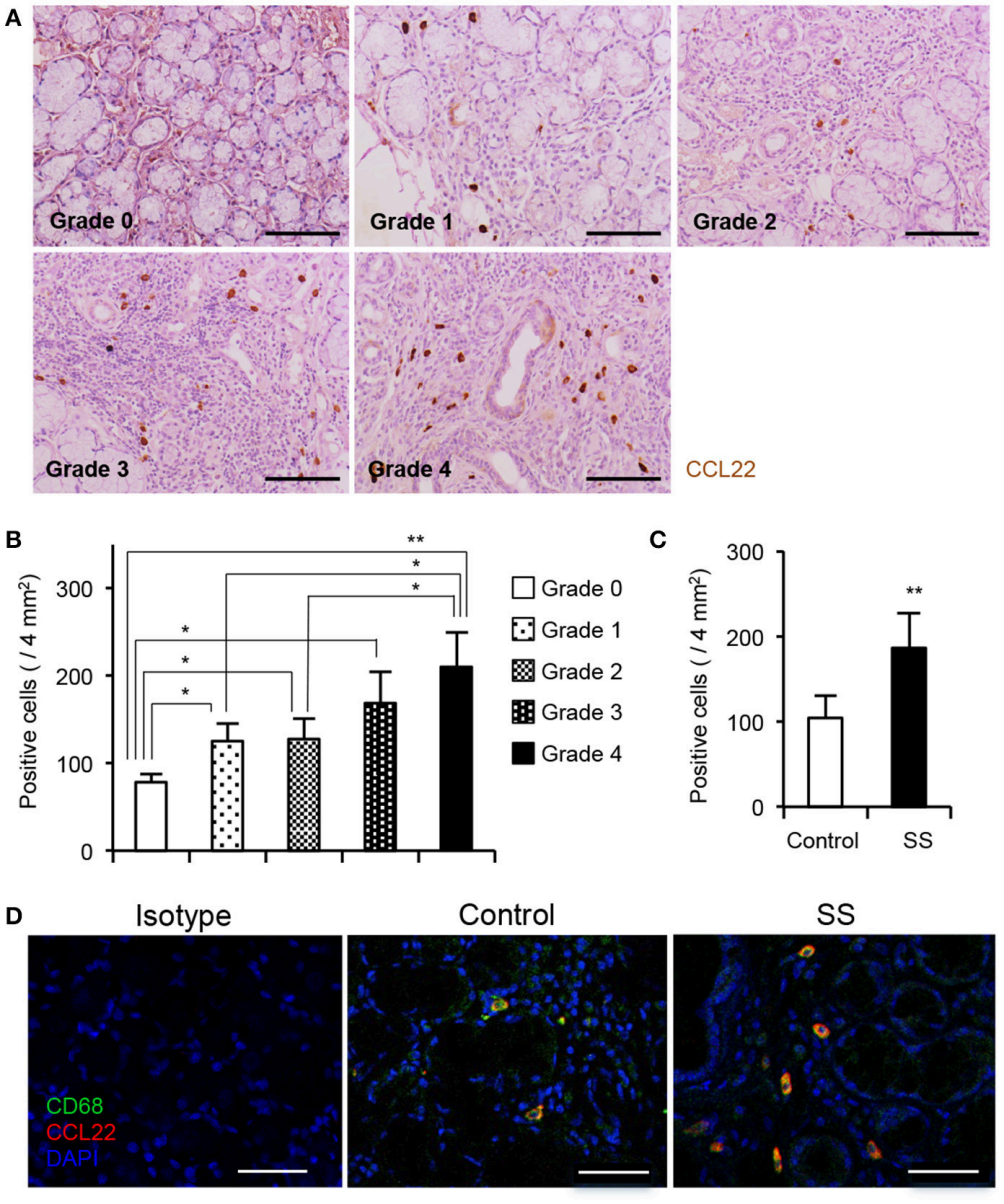
CCL22-producing sMΦs of minor salivary gland tissue from patients with SS. **(A)** CCL22^+^ cells from the minor salivary gland tissues from controls and patients with SS were detected by immunohistochemical analysis. Sections of controls and patients with SS (Grade 1 to 4) were used for analysis. Photos are representative of 5–7 samples in each group. **(B)** The number of CCL22^+^ cells was determined, and data are presented as mean ± SEM of five samples. *p < 0.05, **p < 0.005 by one-way ANOVA with Turkey's multiple comparison post-test. **(C)** Correlation in CCL22^+^ cells between focus score 1 < and 1 ≧ patients. The number of CCL22^+^ cells was determined, and data are presented as mean ± SEM of five samples. *p < 0.05, **p < 0.005 by Student's *t*-test. **(D)** CCL22-producing CD68^+^ sMΦs were detected by confocal analysis using the section of controls and SS patients (Grade 4). Photos are representative of 5 samples in each group. Bar: 50 μm.

To determine whether the CCL22-producing sMΦs contribute to autoimmune lesions in patients with SS, immunohistochemical analysis was performed with anti-CCL22 Ab using minor salivary gland tissues obtained from controls and patients with SS. Based on the degree of lymphocyte infiltration, tissue sections of lip biopsy specimens were divided into four grades (23, 28). Numerous CCL22-producing cells were detected in the high-grade biopsy sections (Figure 6A). Pathological grade and the number of CCL22-producing cells were significantly correlated (Figure 6B). The histopathological criterion of SS diagnosis was a focus score ≧1 which includes Grade 3 and 4 (23). Compared with patients with a focus score < 1, the number of CCL22^+^ cells was significantly higher in the salivary gland tissues from patients with SS with a focus score ≧1 (Figure 6C). In addition, confocal microscopy revealed many CCL22-producing CD68^+^ sMΦs in the minor salivary gland tissues from patients with SS (Grade 4); however, only few CCL22^+^ sMΦs were detected in controls (Figure 6D). In addition, CCL22 was not expressed in stromal cells, such as fibroblasts, endothelial cells, and nerve cells (Supplemental Figure 4A). Furthermore, confocal microscopic analysis confirme that Keratin^+^ epithelial cells (Supplemental Figure 4B), CD3^+^ T cells (Supplemental Figure 4C), CD19^+^ B cells (Supplemental Figure 4D), and S100^+^ DCs (Supplemental Figure 4E) did not express CCL22. A previous report indicated that CCL22 is secreted by macrophages or DCs *in vitro* and *in vivo* (29). This result indicates that CCL22-producing sMΦs play a key role in the formation of autoimmune lesions in the target organ of patients with SS.

## Discussion

In the current study, we investigated the relationship between autoimmunity and MΦs in the target organs in the SS mouse model and the patient with SS. Resident MΦs were divided into two subsets that displayed low and high expression levels of CD11b in the SS model mice. Via the control of T cell migration and cytokine production, CCL22-producing CD11b^high^ macrophages play a key role in the development of autoimmune lesions in the salivary glands.

The number of MΦs in the salivary glands was significantly higher in the SS model mice than in the control mice at 8 weeks of age; however, no difference was observed in the respect at the subsequent time-point. The number of CD11b^low^ sMΦs also significantly increased at 8 weeks of age, which is the stage of onset of autoimmune lesions in the model. In contrast, the number of CD11b^high^ sMΦs was significantly higher in the SS model mice than in the control mice between 12 and 16 weeks of age, which corresponds to the stage wherein development of autoimmune lesions occurs in the SS model mice. These results suggest that the phenotypic change to CD11b^high^ sMΦs within the target organ may be induced during the development of autoimmune lesions. In addition, it is possible that bone marrow-derived myeloid cells may accumulate to become MΦs in the target organ.

During various inflammatory processes, naïve monocytes differentiate into pro-inflammatory M1 and anti-inflammatory M2 MΦs (30, 31). The diverse phenotypes and functionality of resident MΦ in different organs is well-documented (32, 33). It is difficult to differentiate between the M1 and M2 subsets based on the surface markers (34). No differences were observed between the control mice and SS model mice with respect to M1/M2 markers. However, expressions of scavenger receptors (CD36 and CD204) on CD11b^low^ sMΦs of the SS model mice were clearly enhanced. Phagocytic activity of both CD11b^low^ and CD11b^high^ sMΦs in the SS model mice was also upregulated. These results indicate that the phenotypic difference between CD11b^low^ and CD11b^high^ does not contribute to the differentiation into M1/M2 MΦ subsets and the phagocytic function of MΦ. On the other hand, it is possible that CD11b^low^ sMΦs may play a role in the pathogenesis of the SS model mice. As shown in Figure 1, the number of CD11b^low^ sMΦs in SS model mice at 8 weeks of age was significantly higher than that in control mice; therefore, the CD11b^low^ sMΦs may contribute to the onset or early stage of the disease. However, further study is required for a detailed analysis of this population in detail in the next project.

We have checked many markers of macrophage in preliminary experiments before focusing on CD11b expression. Among these, we found that CD11b expression in the target tissue of SS model mice changed with disease progression in the SS model mice. Therefore, we decided to focus on the analysis of the role or function of CD11b^low/high^ sMΦs in the target tissues at the onset or development of the disease.

To determine a key molecule for the development of autoimmune disease in the SS model mice, experiments were conducted in two steps. The first step was comprehensive gene analysis limiting chemokine genes to compare the gene expression of CD11b^high^ sMΦs with that of CD11b^low^ sMΦs in the SS model mice. In the second step, several genes among the upregulated genes that showed a significant difference between the control and SS model mice were picked up. Among these, the CCL22 gene of CD11b^high^ sMΦs was selected as a potential candidate that plays a key role in the development of autoimmune lesions. Alongside the development of autoimmune lesions, CCL22-producing CD11b^high^ sMΦs were increased in SS model mice. M2a, a subset of M2 MΦ, induced by IL-4 and IL-13 exposure expresses arginase I and produces CCL22, IL-10, TGF-β, IL-1Ra, CCL17, and CCL24 to promote Th2 cells, eosinophils, and basophils (1, 2, 3, 4, 6). The CCL22-producing sMΦs seem to differ from the M2a MΦ subset.

CCL22 is one of the C-C motif chemokines and is termed as MΦ-derived chemokine (MDC) in humans and mice. CCL22 and CCL17 bind to CCR4, and both chemokines are 39% identical at the amino acid level (34). Both CCL22 and CCL17 are highly expressed in the thymus (35, 36). The role of CCL22 in peripheral T cells is not clear. IFN-γ-producing Th1 cells were shown to contribute to the onset of autoimmune lesions in the SS model mice (24). In the present study, cytokine production in CD4^+^ T cell response to CCL22 showed differences between the spleen, cLN, and target tissue in the SS model mice. It is possible that the differences regarding receptor expression may be related to the cytokine production through different signaling of the T cells. Hence, CCL22 may play a key role in the breakdown of local immune tolerance in the target organ to induce autoimmune lesions in SS. In addition, our hypothesis is that CCL22 from CD11b^high^ sMΦs may enhance the migration of effector T cells into the target organ through CCR4 on T cells and also the cytokine production, such as IFN-γ, by T cells. In addition, the mRNA expressions of *IFN-*γ, *IL-4*, and *IL-17* in T cells isolated from the salivary gland tissues of SS model mice were enhanced by CCL22. IFN-γ and IL-4 protein secretion was also enhanced by CCL22. As for the discrepancy in IL-17 between the mRNA and protein expressions of IL-17, protein secretion of IL-17 may be influenced by any other factor. Previous reports demonstrate that CCR4 is predominantly expressed by Th2 cells, cutaneous lymphocyte antigen-positive skin-homing T cells, and Treg cells (36, 37). Therefore, CCL22 is considered one of the Th2-associated chemokines (38, 39). Our result was consistent with the elevated CCL22 in salivary gland tissues from patients with SS as described previously (40). The high expression level of CCR4 on CD4^+^ T cells that infiltrate the target organ is a novel finding that highlights the key role of the CCL22-CCR4 axis in the autoimmune reaction in the target organ. Previously, CCL22 was detected around the ductal epithelial cells, whereas CCR4 was detected on infiltrating lymphocytes in the minor salivary glands of patients with SS. From our study, any subset of sMΦs may be the source of CCL22 in the target organ of SS. Th1 and Th17 cells may be involved in the initiation of SS, and Th2 cells may contribute to disease progression through the interaction between chemokines and chemokine receptors, such as CCL22 and CCR4. In contrast, although it was reported that CCL22 gene expression of minor salivary glands of patients with SS was less pronounced (20), our study demonstrated that a part of macrophage subsets highly produces CCL22 in the target tissue in SS. Therefore, it is possible that the increased gene expression of CCL22 in the whole tissue cannot be observed. We determined the concentration of CCL22 for *in vitro* migration assay based on a previous report (41) and our preliminary experiment (Supplemental Figure 2). Indeed, 200 ng/mL of CCL22 may be much higher than the physiological concentration. However, it is difficult to determine the physiological or pathological concentration *in vivo*, and the concentration gradient may change with disease progression in the SS model mice.

In the current study, anti-CCL22 Ab has a therapeutic effect on the autoimmune lesions in the SS model mice. A previous report demonstrated that CCL22 regulates experimental autoimmune encephalaomyelitis (EAE) via the control of MΦ chemoattraction and effector function (27, 42, 43). CCR4 is also known to play a potent role in the development of EAE (44, 45). Furthermore, a CCR4 antagonist was shown to ameliorate EAE via the inhibition of Th1 and Th17 polarization of antigen-induced T cell response (46, 47). In contrast, CCL22-mediated recruitment of Treg cells to the pancreas protects against autoimmune diabetes in a murine type 1 diabetes model (48, 49). CCL22 has also been implicated in various diseases, including allergic disease, and lymphoma (50–54). CCL22 and its receptor contribute to the onset or development of immune disorders by inducing changes in the expression and contribution or the functions (55, 56). In our model, CD11b^high^ sMΦs intensively produced CCL22 to influence T cell responses in the target tissue. In addition, as the infiltration of CD8^+^ T cells in the salivary gland tissues was also suppressed by the injection of anti-CCL22 Ab, the same mechanism may apply for CD8^+^ T cell migration to the target organ. A large number of CD8^+^ T cells were accumulated in the salivary gland tissue of SS model mice (Figure 5D). As mentioned, CD4^+^ T cells are the main subset of immune cells infiltrated in the salivary gland in this model in the early stage. As shown in Figure 5, we analyzed the mice at 10 weeks of age, when CD8^+^ T cells also are infiltrated in addition to the other immune cell populations.

We analyzed the cytokine mRNA expression of cultured T cells *in vitro* to examine the direct effect of CCL22 on T cells. It is important to detect cytokine expression directly in the target tissues. However, it is difficult to assess the effect of CCL22 on the cytokine expression of T cells *in vivo*. Analysis of CD11b-conditional CCL22 gene knockout mouse in the next study will help define the *in vivo* function of CCL22 in autoimmunity.

In this study, in addition to CCL22 gene, several chemokine genes of CD11b^high^ sMΦs were upregulated, suggesting that complicated chemokine network by resident MΦs with phenotypic change affects the pathogenesis of autoimmune lesions. Moreover, CCL22 gene expression of lung in the SS model mice was significantly higher than that of control mice. As slight inflammatory lesions of the lung in the SS model mice are observed with age, CCL22 may play a key role in the pulmonary lesions.

To summarize, a phenotypic change in the resident sMΦs of SS model mice was observed during the development of autoimmune lesions. CCL22-producing resident MΦs influence T cell migration and cytokine production in the target organ of the SS model mice. Interventions that target MΦs may serve as a potential novel treatment for autoimmune diseases.

## Ethics statement

All animal experiments were reviewed and approved by he Committee on Animal Experiments of Tokushima University and Biological Safety Research Center, Japan (Permit Number: T29-115). Labial salivary gland (LSG) samples were obtained from patients with SS and controls. The study was approved by the Institutional Review Board of the Tokushima University Hospital, Japan (No. 2802). Written informed consent was received from participants prior to inclusion in the study in accordance with the Declaration of Helsinki.

## Author contributions

AU designed and performed the experiments, analyzed the data, and prepared the manuscript. RA designed the experiments and provided intellectual assistance. KO, AY, and TT performed the experiments. YK designed the experiments. KA and MA prepared human samples. NI provided broad guidance in experimental design, data analysis, and manuscript preparation. All authors read, reviewed, and approved the final manuscript.

### Conflict of interest statement

The authors declare that the research was conducted in the absence of any commercial or financial relationships that could be construed as a potential conflict of interest.

## Supplementary material

Supplemental Figure 1 Gating strategy and representative gating of immune cells obtained from salivary gland tissues in the SS models. FSC-A/SSC-A, FSC-A/FSC-H, and FSC/CD45.2 panels are shown. After gating single cells and CD45.2^+^ cells among live cells, 7-AAD^−^CD3^−^CD19^−^ cells were gated for obtaining MΦ population.

Supplemental Figure 2 **(A)** mRNA expressions of CXCR3A, CCR3, and CX3CR1 in salivary gland tissues from control and SS model mice at 12 weeks of age were determined by qRT-PCR. Data are presented as mean ± SEM. *n* = 5. The primer sequences used were as follows: CXCR3A: forward, 5′-TGCTAGATGCCTCGGACTTT-3′, and reverse, 5′- CGCTGACTCAGTAGCACAGC-3′, CCR3: forward, 5′-TTGATCCTCATAAAGTACAGGAAGC-3′, and reverse, 5′- CAATGCTGCCAGTCCTGCAA-3′, CX3CR1: forward, 5′- CACCATTAGTCTGGGCGTCT-3′, and reverse, 5′- GATGCGGAAGTAGCAAAAGC-3′. Relative mRNA expression of each transcript was normalized against β-actin mRNA. **(B)** Migratory activity of CD4^+^ T cells from SS model mice to CCL22 (0, 50, 100, and 200 ng/mL) was analyzed by *in vitro* migration assay using trans-well. Data are representative of three independent experiments. ^*^*p* < 0.05 by Student's *t*-test.

Supplemental Figure 3 **(A)** Intracellular expressions of IFN-γ, IL-4, and IL-17 of activated splenic CD4^+^ T cells. CD4^+^ T cells purified from spleen of the control and SS model mice were stimulated with PMA (50 ng/mL) and ionomycin (1 μg/ml) for 6 h with Golgi-stop. Data are representative of three independent experiments. **(B)** CD4^+^ T cells purified from the spleen in control mice were cultured with CCL22 (200 ng/mL) for 6 h. The mRNA expressions of IFN-γ, IL-4, and IL-17 were detected by qRT-PCR. Data are presented as mean ± SEM relative to that without CCL22 and are representative of three independent experiments. ^*^*p* < 0.05 by Student's *t*-test. **(C)** Intracellular IFN-γ expression in CD4^+^ T cells of SS model mice. CD4^+^ T cells were stimulated with or without CCL22 (200 ng/mL) in the presence of anti-CD3/CD28 mAb for 48 h, and were subsequently cultured with PMA (50 ng/mL) and IM (1 μg/mL) in the presence of Brefeldin A for the last 6 h. The results are representative of five samples in each group. **(D)** Intracellular cytokine expressions of IFN-γ, IL-4, and IL-17 in splenic CD4^+^ T cells of control and SS model mice were analyzed by flow cytometer. Data are presented as mean ± SEM relative to that without CCL22. *n* = 5. ^*^*p* < 0.05 by Student's *t*-test, ^**^*p* < 0.005 by Student's *t*-test.

Supplemental Figure 4 **(A)** Stromal cells did not express CCL22. CCL22 was detected by immunohistochemical analysis. Bar: 500 and 50 μm. **(B–E)** Confocal microscopic analysis of CCL22 expression of Keratin^+^ epithelial cells **(B)**, CD3^+^ T cells **(C)**, CD19^+^ B cells **(D)**, and S100^+^ DCs **(E)** using the sections of SS patients (Grade 4). Photos are representative of five samples in each group. Bar: 50 μm.
