# Supplementary figures and images for: CCL22-Producing Resident Macrophages Enhance T Cell Response in Sjögren's Syndrome
Source: Front Immunol. 2018 Nov 8;9:2594. doi: 10.3389/fimmu.2018.02594 (PMC6236111; doi:10.3389/fimmu.2018.02594)

## Supplementary Figure 1

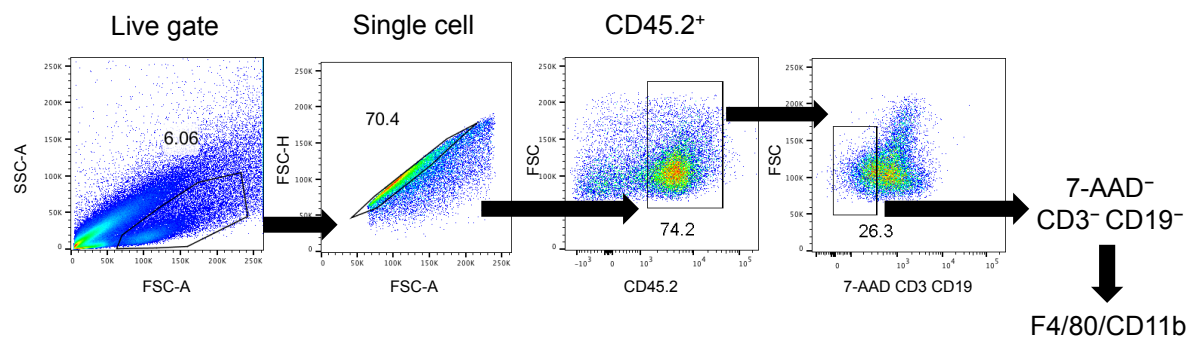

Supplemental Figure 2

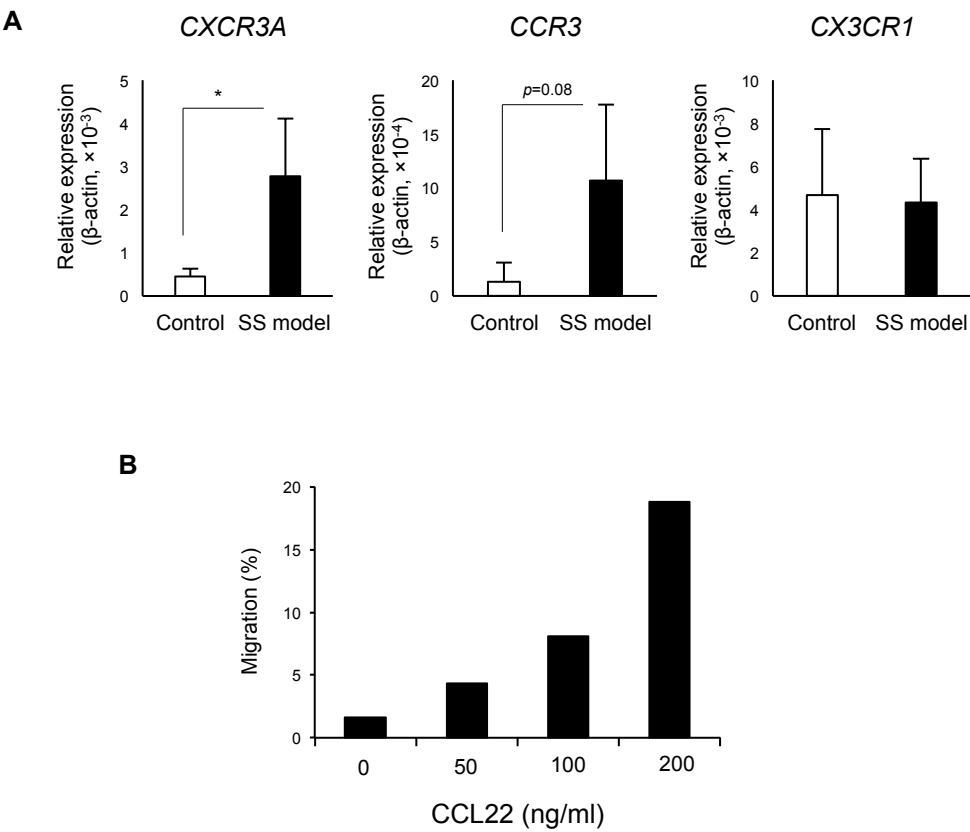

Supplemental Figure 3

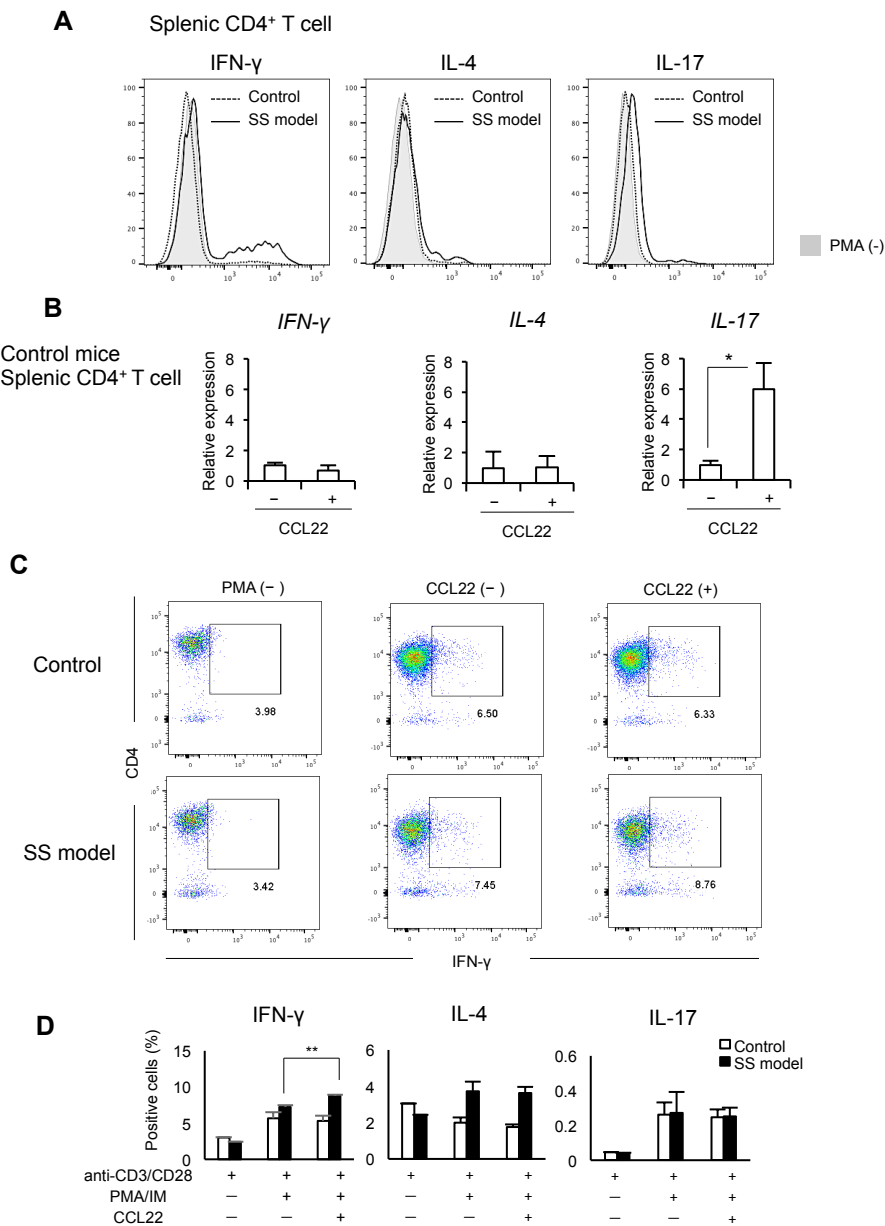

Supplemental Figure 4

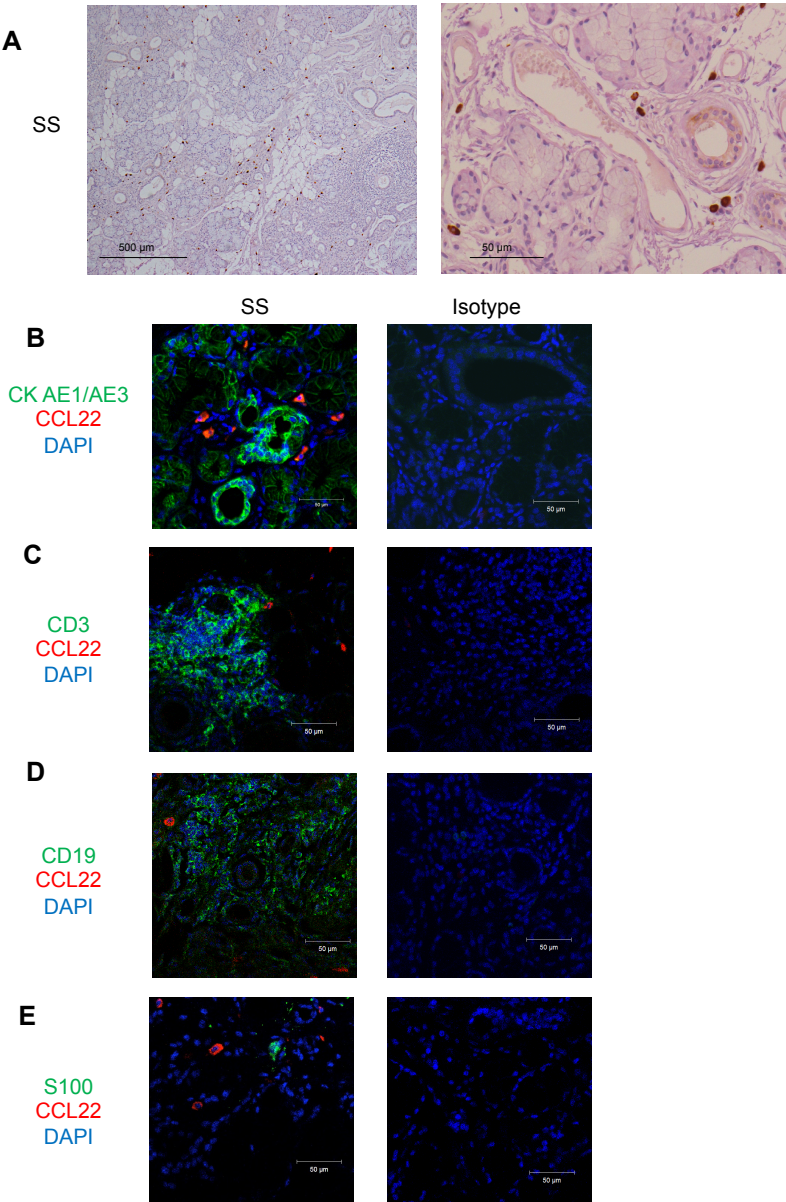

Supplement: Supplemental Figure 1 — Gating strategy and representative gating of immune cells obtained from salivary gland tissues in the SS models. FSC-A/SSC-A, FSC-A/FSC-H, and FSC/CD45.2 panels are shown. After gating single cells and CD45.2+ cells among live cells, 7-AAD−CD3−CD19− cells were gated for obtaining MΦ population. [file Data_Sheet_1.PDF]
